# Biomarker advancements in cerebral small vessel disease: An overview

**DOI:** 10.4103/NRR.NRR-D-24-01329

**Published:** 2025-05-06

**Authors:** Wenqian Luo, Chenhui Cao, Wenli Li, Ting Wei, Zeyu Zhao, Guanqing Wang, Xiaoli Li, Yanbin Li, Bin Liu

**Affiliations:** Department of Neurology, The First Affiliated Hospital of Shandong First Medical University & Shandong Provincial Qianfoshan Hospital, Shandong Institute of Neuroimmunology, Jinan, Shandong Province, China

**Keywords:** aging, biomarkers, cerebral small vessel disease, cerebrospinal fluid, endothelial dysfunction, genes, inflammatory biomarkers, neurovascular unit

## Abstract

Cerebral small vessel disease is a condition caused by chronic cerebral hypoperfusion due to microvascular damage and is a major contributor to stroke and dementia. Traditionally, its diagnosis has relied primarily on neuroimaging findings. However, recent advances in the understanding of cerebral small vessel disease pathophysiology have opened new avenues for early detection and targeted therapeutic interventions. Notably, the identification and investigation of cerebral small vessel disease–related biomarkers have emerged as a promising strategy for early diagnosis. This review provides an overview of recent research on cerebral small vessel disease biomarkers, including plasma biomarkers, cerebrospinal fluid biomarkers, and genetic markers. Finally, we discuss future directions and trends in the clinical validation of these biomarkers.

## Introduction

Cerebral small vessel disease (CSVD) encompasses a group of disorders characterized by brain parenchymal damage resulting from pathological changes in cerebral microvessels, including small penetrating arteries, arterioles, venules, capillaries, and the surrounding 2–5 mm radius, as well as vascular structures within the subarachnoid space (Pantoni, 2010; Litak et al., 2020). Damage to these vessels and associated structures leads to a range of pathophysiological alterations, such as arteriolosclerosis, lipohyalinosis, fibrinoid necrosis, cerebral amyloid angiopathy (CAA), perivascular space (PVS) enlargement, microaneurysm formation, blood–brain barrier (BBB) disruption, and vasculitis. These pathological changes contribute to various clinical manifestations and neuroimaging abnormalities, including lacunes, recent small subcortical infarcts, white matter hyperintensities (WMH), cerebral microbleeds (CMBs), PVSs, brain atrophy, cortical superficial siderosis, convexity subarachnoid hemorrhage, and spontaneous intracerebral hemorrhages attributed to small vessel disease (SVD) (Duering et al., 2023). Among these, WMH, CMBs, and lacunar infarctions (LI) are recognized as the hallmark MRI features of CSVD (Gouw et al., 2011).

The clinical manifestations of CSVD are diverse and can be broadly categorized into four types: motor dysfunction, emotional disturbances, cognitive impairment, and urinary and fecal incontinence (Wardlaw et al., 2019). Notably, WMH and CMBs have been shown to correlate with cognitive dysfunction (Debette et al., 2019; Wardlaw et al., 2019) and may also increase the risk of mortality (Debette et al., 2019). However, no standardized diagnostic criteria for CSVD currently exist, and diagnosis is primarily based on clinical presentation and corresponding neuroimaging findings. CSVD can be classified into six subtypes: type I (arteriosclerosis-related CSVD), type II (amyloid-related CSVD), type III (genetic CSVD distinct from amyloid angiopathy), type IV (inflammatory/immunologically mediated CSVD), type V (venous collagenosis-related CSVD), and type VI (other forms, such as radiation-induced CSVD) (Litak et al., 2020). The underlying mechanisms of these subtypes are complex and not yet fully elucidated.

**Figure 1:**
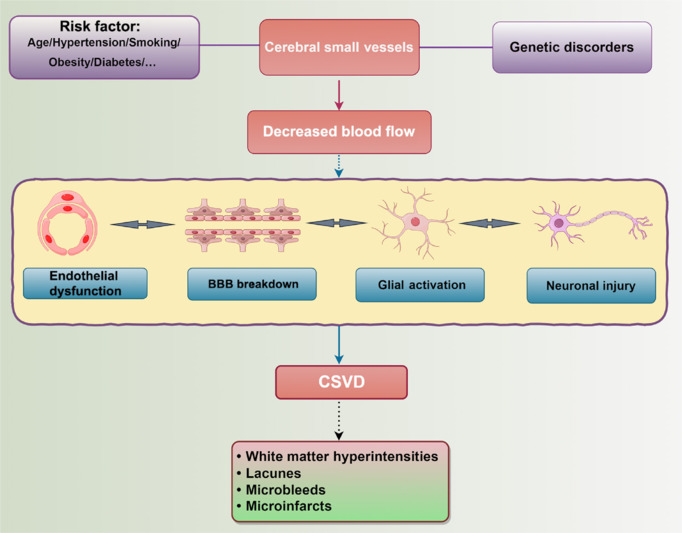
Schematic diagram of key pathophysiological mechanisms underlying CSVD. BBB: Blood–brain barrier; CSVD: cerebral small vessel disease.

Impaired cerebral blood flow can result in chronic cerebral hypoperfusion, triggering a cascade of cellular processes, including endothelial dysfunction, BBB disruption, glial activation, neuronal injury, and subsequent tissue ischemia and hypoxia, all of which contribute to brain parenchymal damage (Hannawi, 2024; **Figure 1**). This review aims to summarize recent research on the relationship between biomarkers and the pathogenesis and progression of CSVD, with an emphasis on advancing early diagnosis and therapeutic strategies.

The identification of reliable biomarkers closely associated with disease progression is crucial for the early diagnosis, prognosis, prevention, and treatment of CSVD-related dementia. At present, treatment and prevention strategies for CSVD remain undefined, and its diagnosis primarily relies on neuroimaging. However, biomarkers capable of detecting CSVD in its early stages have yet to be identified. This review provides an overview of recent advances in biomarker research related to CSVD, highlighting their potential role in improving early detection and guiding therapeutic interventions.

## Search Strategy

We conducted a comprehensive search of the PubMed database for relevant articles published between 1991 and 2024. The search terms included: (leukoaraiosis) OR (white matter lesions) OR (white matter hyperintensities) OR (lacunar stroke) OR (lacunes) OR (perivascular spaces) OR (microbleeds) OR (inflammation) OR (blood–brain barrier) OR (endothelial dysfunction) OR (coagulation) OR (metabolism) OR (aging) OR (NVU injury) OR (gene) OR (CSF) AND (cerebral small vessel disease) OR (CSVD). No language restrictions were applied. Additionally, the references of the retrieved articles were reviewed, and relevant studies were included based on their significance. The final selection of articles was determined by the authors based on relevance to the topic.

## Plasma Biomarkers

**Figure 2:**
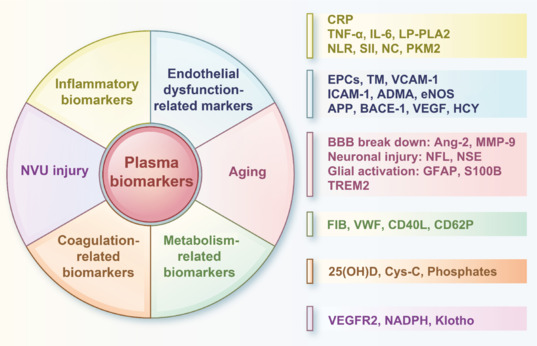
Candidate plasma biomarkers categorized by their role in the pathogenesis of cerebral small vessel disease. 25(OH)D: 25-Hydroxyvitamin D; ADAM: asymmetric dimethylarginine; Ang-2: angiopoietin-2; BACE-1: beta amyloid precursor protein cleavage enzyme-1; CD40L: CD40 ligand; CD62P: platelet-selectin; CRP: C-reactive protein; Cys-C: cystatin C; eNOS: endothelial nitric oxide synthase; EPCs: endothelial progenitor cells; FIB: fibrinogen; GFAP: glial fibrillary acidic protein; HCY: homocysteine; ICAM-1: intercellular cell adhesion molecule-1; IL-6: interleukin-6; LP-PLA2: plasma lipoprotein-associated phospholipase A2; MMP-9: matrix metalloproteinase-9; NADPH: nicotinamide adenine dinucleotide phosphate; NC: neutrophil count; NFL: neurofilament light chain; NLR: neutrophil-to-lymphocyte ratio; NSE: neuron-specific enolase; NVU: neurovascular unit; PKM2: Pyruvate kinase M2; SII: systemic immuno-inflammatory index; TM: thrombomodulin; TNF-α: tumor necrosis factor-α; TREM2: triggering receptor expressed on myeloid cells 2; VCAM-1: vascular cell adhesion molecule-1; VEGF: vascular endothelial growth factor; VEGFR2: vascular endothelial growth factor receptor 2; vWF: von Willebrand factor.

Changes in biomolecules resulting from neuronal or neurovascular damage can be detected in the peripheral circulation. Plasma biomarkers hold promise for the early detection of CSVD due to their accessibility, cost-effectiveness, and rapid assessment. This review summarizes recent research on CSVD-related plasma biomarkers, focusing on those associated with inflammation, endothelial dysfunction, coagulation, neuronal injury, glial cell activation, metabolism, and aging. The monitoring of these biomarkers may facilitate the early identification of CSVD, offering new insights for improving diagnosis and treatment strategies. A summary of the most relevant plasma biomarkers linked to CSVD is presented in **Figure 2**.

### Inflammatory biomarkers

#### C-reactive protein

Elevated baseline levels of systemic inflammatory markers have been shown to predict the severity and progression of CSVD (Low et al., 2019). Among these, C-reactive protein (CRP) and high-sensitivity CRP (hs-CRP) are the primary systemic inflammatory markers associated with CSVD (Wan et al., 2023). CRP is a sensitive but nonspecific systemic marker of inflammation and tissue damage, primarily regulated by pro-inflammatory cytokines, particularly interleukin-6 (IL-6) (Sproston and Ashworth, 2018). Studies have demonstrated that elevated serum CRP levels, in combination with high IL-6, are associated with an increased risk of vascular dementia (Dziedzic, 2006; Ravaglia et al., 2007; Androsova et al., 2013). A prospective study conducted in an elderly Asian community cohort found that baseline serum CRP levels were associated with the future development of vascular dementia but were not linked to Alzheimer’s disease (AD) after adjusting for common cardiovascular risk factors, stroke, and competing risks of death (Hsu et al., 2017). Additionally, Cao and Sun (2022) reported a negative correlation between serum hs-CRP levels and CSVD-induced cognitive dysfunction, suggesting that hs-CRP may serve as a predictor of CSVD-related cognitive impairment. Serum CRP levels have been correlated with various CSVD imaging markers, including white matter lesions (WMLs), LIs, CMBs, and enlarged PVS (Hilal et al., 2018). Higher hs-CRP levels have been linked to the progression of white matter damage (van Dijk et al., 2005), while CRP levels have also been associated with white matter injury. In an experimental study using a rat model of chronic cerebral hypoperfusion induced by permanent bilateral common carotid artery ligation, Juma et al. (2011) demonstrated a correlation between CRP expression and WML in brain tissue. Furthermore, Koh et al. (2011) found that CRP levels were significantly higher in patients with acute LIs and microbleeds compared to those without microbleeds. However, the relationship between CRP levels and CSVD remains controversial, as some studies have found no significant correlation between CRP levels and CSVD severity (Reitz et al., 2007; Wada et al., 2008). A community-based Austrian stroke prevention study investigating the association between CRP, carotid atherosclerosis (AS), white matter disease, and CSVD severity concluded that CRP levels were not significantly correlated with CSVD severity or progression (Schmidt et al., 2006). The inconsistencies across studies may be attributed to differences in cohort characteristics, sample sizes, and assessment methodologies. While CRP plays a crucial yet nonspecific role as an inflammatory marker, further research is needed to clarify its potential as a reliable biomarker for CSVD and to determine whether CRP and hs-CRP can be effectively used in clinical practice for CSVD risk assessment and monitoring.

#### Tumor necrosis factor-α and interleukin-6

Activated immune cells produce a variety of cytokines, including pro-inflammatory cytokines such as IL-6 and tumor necrosis factor (TNF)-α, as well as anti-inflammatory cytokines such as IL-10. Elevated levels of TNF-α and IL-6 have been frequently observed in patients with CSVD. TNF-α has been implicated in the development of WMHs but has not been associated with CMBs (Li et al., 2020; Cai et al., 2021). Over the years, studies have yielded conflicting findings regarding the relationship between IL-6 and CSVD progression. While some research has reported a negative association between baseline plasma IL-6 levels and MRI-detected CSVD progression over four years (Satizabal et al., 2012), other studies have demonstrated a positive correlation. For instance, IL-6 has been identified as a predictor of new LIs (Staszewski et al., 2018b). In age-related CSVD, early immune cell senescence weakens normal immune responses, increasing susceptibility to infections. This aging process triggers the release of pro-inflammatory cytokines, including IL-1β, IL-6, IL-8, and IL-17, through the senescence-associated secretory phenotype, exacerbating endothelial damage and BBB disruption, thereby contributing to CSVD progression (Del Cuore et al., 2022). While IL-6 and TNF-α are strongly associated with the progression of CSVD (Zhang et al., 2022a), emerging research highlights the crucial role of anti-inflammatory cytokine regulation. In particular, the downregulation of IL-10 has been implicated in CSVD pathogenesis, suggesting that an imbalance between pro- and anti-inflammatory cytokines may play a significant role in disease progression (Jian et al., 2020).

#### Plasma lipoprotein-associated phospholipase A2

Lipoprotein-associated phospholipase A2 (Lp-PLA2) is an enzyme secreted by circulating macrophages that plays a crucial role in the inflammatory response and is widely recognized as a marker of neuroinflammation. The involvement of Lp-PLA2 in AS has been well documented (Ballantyne et al., 2005). Serum Lp-PLA2 levels have been linked to CMBs caused by CSVD, with elevated plasma Lp-PLA2 levels being closely associated with an increased number of CMBs (Romero et al., 2012). Additionally, Lp-PLA2 has been identified as an independent predictor of cognitive impairment in patients with CSVD. It may provide valuable information for clinicians in assessing the severity of the disease and the extent of cognitive decline associated with CSVD (Zhu et al., 2019). As a result, Lp-PLA2 is emerging as a potential therapeutic target for preventing cognitive impairment in patients with CSVD.

#### Neutrophil-to-lymphocyte ratio, systemic immuno-inflammatory index, and neutrophil count

Neutrophil-to-lymphocyte ratio (NLR) and systemic immuno-inflammatory index (SII) are reliable indicators of immune status and are associated with the progression of vascular diseases, making them strong prognostic markers. NLR serves as a predictor of subclinical inflammation, while SII reflects systemic immune status. In a community-based aging study, Chuang et al. (2023) found a strong positive correlation between NLR and CSVD, suggesting that NLR could be a predictor of the disease. Another study demonstrated that SII is linked to a higher risk of MRI markers of CSVD, such as WMHs and enlarged PVSs, but not CMBs (Jiang et al., 2022). An elevated neutrophil count (NC) in the blood indicates a greater total CSVD burden, with higher NC levels being associated with more LIs and larger PVSs. These studies indicate that NLR, NC, and SII independently correlate with stroke incidence, disease severity, adverse outcomes, and CSVD severity. Research by Hou et al. (2022) further suggests that higher NLR in CSVD patients may be linked to an increased risk of mild cognitive impairment. While NLR, SII, and NC are associated with many neurological diseases and play significant roles in CSVD progression, current research indicates that these markers are still challenging to use as specific biomarkers for CSVD. Further investigation is needed to establish their reliability and clinical applicability in CSVD diagnosis and management.

#### Pyruvate kinase M2

Pyruvate kinase M2 (PKM2) is a crucial rate-limiting enzyme in glycolysis and plays a role in various physiological processes, including inflammatory responses, cell apoptosis and proliferation, and oxidative stress. PKM2 is predominantly expressed in astrocytes and other glial cells in the nervous system (Wei et al., 2020; Zhang et al., 2024b) and is implicated in a range of diseases. PKM2 influences neutrophil overactivation following cerebral ischemia by enhancing STAT3 phosphorylation, which leads to thrombus-driven inflammation and exacerbates stroke severity (Dhanesha et al., 2022). Under hypoxic conditions, PKM2 also promotes the production of amyloid-β (Aβ), worsening cognitive impairment in AD (Han et al., 2021; Pan et al., 2022). Additionally, PKM2 facilitates the proliferation and migration of vascular smooth muscle cells (SMCs), thereby contributing to the progression of AS (Zhao et al., 2020). Plasma PKM2 levels have been proposed as a marker of vascular inflammation, offering potential for disease diagnosis and monitoring (Esen et al., 2022). Inflammation plays a pivotal role in the development of CSVD. Bian et al. (2023) demonstrated that serum PKM2 levels are positively correlated with MRI markers of CSVD, such as WMHs and enlarged PVSs, and negatively correlated with cognitive decline in CSVD patients.

Inflammation may contribute to CSVD through various mechanisms. While these biomarkers show an association with CSVD imaging findings, their specificity remains a topic of debate. However, they are easily detectable in clinical practice. A summary of the inflammatory biomarkers associated with MRI features of CSVD is presented in **Additional Table 1**.

**Additional Table 1:** Associations of systemic inflammation biomarkers with MRI features of CSVD

Blood biomarker	Neuroimaging Marker	Study population	Reference
C-reactive protein and highly sensitive C-reactive protein	WMH, lacunar infarcts	1033 older participants aged 60-90 yr	van Dijk et al., 2005
tumor necrosis factor-α and interleukin-6	WMH	960 participants aged > 35 yr	Zhang et al., 2022a
Plasma lipoprotein-associated phospholipase A2	Cerebral microbleeds	819 participants aged ≥ 65 yr	Romero et al., 2012
Neutrophil-to-lymphocyte ratio, systemic immuno-inflammatory index, and neutrophil count	Enlarged perivascular space, lacunar stroke, WMH	3052 participants aged 50-75 yr	Jiang et al., 2022
Pyruvate kinase M2	WMH, enlarged perivascular space	219 patients with CSVD	Bian et al., 2023

CSVD: Cerebral small vessel disease; WMH: white matter hyperintensity.

### Endothelial dysfunction–related biomarkers

Vascular endothelial cells (VECs) play a crucial role in the onset and progression of CSVD, particularly in the development of WMHs, LIs, and CMBs. Endothelial dysfunction is widely recognized as a key pathogenic mechanism driving the development of CSVD. Biomarkers associated with endothelial dysfunction are often linked to cardiovascular diseases (CVDs), which may reduce their specificity in the context of CSVD. However, these biomarkers may still offer predictive value regarding the progression of CSVD. A summary of the endothelial dysfunction-related biomarkers associated with MRI features of CSVD is presented in **Additional Table 2**.

**Additional Table 2:** Association of endothelial injury biomarkers with MRI features of CSVD

Blood biomarkers	Neuroimaging markers	Study population	Reference
Endothelial progenitor cells	CSVD burden	64 older adults without dementia or stroke and aged > 55-90 yr	Kapoor et al., 2021
Thrombomodulin	WMH, lacunar infarcts	110 patients with previous lacunar stroke and 50 community controls	Hassan et al., 2003
Vascular cell adhesion molecule-1	WMH, lacunar infarcts	110 Alzheimer's disease patients and 50 age- matched controls; 30 patients with lacunar stroke and 37 healthy controls	Brown et al., 2015; Huang et al., 2015;
Intercellular cell adhesion molecule-1	WMH, lacunar infarcts	1763 participants from the Framingham Offspring cohort	Shoamanesh et al., 2015
Asymmetric dimethylarginine	WMH	35 CSVD patients and 35 controls	Janes et al.,2019
Endothelial nitric oxide synthase	lacunar infarcts	300 CSVD patients and 600 controls	Hassan et al., 2004b
Beta amyloid precursor protein cleavage enzyme-1	CMBs		Gong et al.,2019
Vascular endothelial growth factor	CMBs, WMH	66 patients with cerebral large artery disease; 80 CSVD patients and 39 controls	Ogata et al., 2019; Manukjan et al., 2023
Homocysteine	WMH, lacunar infarcts	172 CSVD patients and 172 controls; 1023 participants aged > 35 yr	Hassan et al., 2004a; Cao et al., 2021

CMBs: Cerebral microbleeds; CSVD: cerebral small vessel disease; WMH: white matter hyperintensity.

#### Endothelial progenitor cells

Endothelial progenitor cells (EPCs) are a heterogeneous population of cells in various stages of maturation (Kapoor et al., 2021). EPCs have the capacity to mature into endothelial cells and self-renew, enabling them to repair blood vessels, promote re-endothelialization, and support angiogenesis. In addition, EPCs can reduce inflammation, promote neuron survival, and maintain myelin integrity in damaged tissues through the release of various factors (Custodia et al., 2022). While most research has focused on the relationship between EPCs and stroke, suggesting that EPCs may enhance recovery and reduce brain damage after a stroke (Sobrino et al., 2007; Rakkar et al., 2020; Custodia et al., 2022), studies have also linked higher EPC levels to a greater burden of CSVD. Kapoor et al. (2021) proposed that elevated EPCs may represent a reactive response to repair vascular endothelial damage, indicating that EPC activation could be an early attempt to restore endothelial function in CSVD. If this hypothesis is confirmed, EPCs might serve as a predictive marker for early-stage CSVD. Some evidence suggests that depletion of the EPC pool in later stages may correlate with progressive cognitive decline (Brenner et al., 2010). However, it is important to recognize that while EPCs contribute to repair and remodeling processes, their production, particularly during these phases, can compromise endothelial barrier integrity and cell junction function. This process may increase inflammation and BBB permeability, potentially exacerbating CSVD (Kapoor et al., 2021). Therefore, whether the mobilization of EPCs in CSVD is ultimately beneficial or harmful requires further investigation.

#### Thrombomodulin

Thrombomodulin (TM) is an endothelial glycoprotein widely expressed in all blood vessels, where it works in concert with thrombin to regulate the activation of protein C, thereby exerting antithrombotic and anti-inflammatory effects (Pescini et al., 2017). Under pathological conditions, elevated concentrations of soluble TM may reflect the degree of endothelial cell injury (Demeulenaere et al., 2018). Numerous studies have reported increased TM levels in individuals with CSVD (Giwa et al., 2012), with these elevated levels being associated with WMLs and LIs (Hassan et al., 2003; Knottnerus et al., 2009). However, Pescini et al. (2017) demonstrated no significant association between TM levels and cerebral autosomal dominant arteriopathy with subcortical infarcts and leukoencephalopathy (CADASIL). While TM is linked to sporadic CSVD, these findings suggest that TM may not be the most suitable biomarker for CSVD (Pescini et al., 2017). Nonetheless, elevated TM levels remain a reliable indicator in specific pathological contexts.

#### Vascular cell adhesion molecule-1

The expression of vascular cell adhesion molecule-1 is induced on endothelial cells and plays a significant role in the pathogenesis of AS. Circulating concentrations of vascular cell adhesion molecule-1 (VCAM-1) serve as biomarkers for the severity of WMLs in vascular pathologies (Huang et al., 2015). As a key member of the cell adhesion molecules (CAMs) family, VCAM-1 is upregulated during endothelial cell activation and mediates leukocyte adhesion, thereby contributing to inflammatory processes. VCAM-1 has been recognized as a biomarker of endothelial dysfunction (Huang et al., 2015; El Husseini et al., 2020) and is associated with CVDs (Castillo et al., 2009; Blanco et al., 2010; Hoyte et al., 2010). Elevated VCAM-1 levels have been observed in the blood of patients with CSVD (Rouhl et al., 2012; El Husseini et al., 2020), with strong correlations to WMLs (Gimenez et al., 2004; Huang et al., 2010, 2015; Zhang et al., 2022a) and LIs (Kozuka et al., 2002; Brown et al., 2015). Recent evidence also suggests a potential link between VCAM-1 levels and vascular cognitive impairment (Hansra et al., 2024). Despite these associations, further research is required to determine the viability of VCAM-1 as a reliable biomarker for CSVD.

#### Intercellular cell adhesion molecule-1

Intercellular cell adhesion molecule-1 (ICAM-1), expressed on VECs, is a key protein that mediates leukocyte adhesion to participate in inflammatory responses (Hassan et al., 2003; Hsu et al., 2011). ICAM-1, as a marker of endothelial damage, is closely associated with various diseases, including multiple sclerosis (Blezer et al., 2015), AS (Nageh et al., 1997; Fassbender et al., 1999), AD (Otgongerel et al., 2023), and stroke (Gregory et al., 2019). Given that endothelial dysfunction is one of the pathogenic mechanisms of CSVD, elevated blood levels of ICAM-1 have been found to correlate with the progression of LIs (Knottnerus et al., 2009; Shoamanesh et al., 2015) and WMLs (Markus et al., 2005; Han et al., 2009; Umemura et al., 2011; Shoamanesh et al., 2015). A previous study has pinpointed ICAM-1 as an independent risk factor for CSVD (Ma et al., 2022). Furthermore, ICAM-1 has been robustly linked to vascular dementia (Ewers et al., 2010; Umemura et al., 2011; Gregory et al., 2019; Hansra et al., 2024). However, whether blood levels of ICAM-1 can be used to predict the progression of CSVD requires further investigation.

#### Asymmetric dimethylarginine

Asymmetric dimethylarginine (ADMA) is an endogenous inhibitor of nitric oxide (NO) synthase that impairs NO production, leading to endothelial dysfunction. As a biomarker of endothelial impairment, ADMA has been shown to increase vascular stiffness and reduce cerebral perfusion, thereby contributing to ischemic brain injury (Kielstein et al., 2006). Elevated ADMA levels have been strongly associated with various cardiovascular and cerebrovascular diseases, including stroke (Yoo and Lee, 2001; Bima et al., 2024), AS (Furuki et al., 2007; Notsu et al., 2015), AD (Selley, 2003), diabetes (Krzyzanowska et al., 2007), and dilated cardiomyopathy (Anderssohn et al., 2012). Given its involvement in these conditions, ADMA is recognized as a predictor of cardiovascular events (Schnabel et al., 2005; Wanby et al., 2006; Choi et al., 2020). With advancing research on CSVD, the link between ADMA and CSVD is becoming increasingly clear. In CADASIL, a genetic form of CSVD, researchers have reported significantly higher ADMA levels in patients compared to controls (Rufa et al., 2008). In sporadic CSVD, ADMA levels have been correlated with WMLs (Khan et al., 2007; Janes et al., 2019) and cognitive impairment (Gao et al., 2015). However, the relationship between ADMA and other imaging markers of CSVD remains to be further explored in future studies.

#### Endothelial nitric oxide synthase

Endothelial NO synthase (eNOS) is a crucial enzyme responsible for maintaining vascular homeostasis. It synthesizes NO from L-arginine in endothelial cells, regulating vascular tone and local blood flow (Katusic and Austin, 2014). NO, produced by endothelial cells, serves as a key molecule linking the cerebral vasculature to neuronal function. The loss of NO is central to endothelial dysfunction (Katusic and Austin, 2014), contributing to vascular stiffening, vasoconstriction, upregulation of leukocyte adhesion molecules, platelet aggregation, and vascular SMC proliferation (Jung et al., 2013; Emdin et al., 2018), all of which are implicated in cognitive decline (Austin et al., 2013; Katusic and Austin, 2014; Tan et al., 2015). Deficiency in eNOS has been associated with various cardiovascular and cerebrovascular diseases, including thrombosis (Tan et al., 2015), AS (Kuhlencordt et al., 2001), AD (Austin et al., 2010; Bagi et al., 2022), traumatic brain injury (Schwarzmaier et al., 2015), microvascular spasm (Lenz et al., 2021), and stroke (Zhang et al., 1996; Cui et al., 2009). The loss of NO increases BBB permeability (Wardlaw et al., 2013), potentially leading to white matter damage (Liao et al., 2021). eNOS has been shown to exert protective effects against CSVD, although this effect has primarily been observed in LIs (Hassan et al., 2004b). In recent years, eNOS-deficient mice have been used as a model for age-related CSVD, providing a valuable tool for studying disease mechanisms (Liao et al., 2021). Enhancing eNOS activity in the vascular endothelium may offer promising preventive strategies against CSVD (McCarty, 2015). Additionally, increasing L-arginine levels has been proposed as a potential approach to mitigate CSVD-related cognitive impairment (Dobrynina et al., 2023).

#### Beta-site amyloid precursor protein cleaving enzyme 1

Beta-site amyloid precursor protein (APP) cleaving enzyme 1 (BACE-1) is a key enzyme involved in APP processing, leading to the production of neurotoxic Aβ. This enzyme is expressed in endothelial cells of the BBB and is implicated in the pathogenesis of CAA (Devraj et al., 2016). In cardiovascular and cerebrovascular diseases, BACE-1 mediates endothelial dysfunction and increases BBB permeability by inhibiting eNOS activation (Zhou et al., 2022). Dysregulation of APP has been identified as a pathological marker of CMBs (Gong et al., 2019). As a critical enzyme in APP processing, BACE-1 is strongly associated with CMBs and plays a pivotal role in cognitive impairment (Gong et al., 2019; Zuliani et al., 2020; Basak et al., 2023). Beyond its role in vascular amyloidosis and endothelial damage, BACE-1 is significantly upregulated in patients with ischemic stroke, where it facilitates inflammatory responses (Bulbarelli et al., 2012). BACE-1 may contribute to CSVD by promoting small vessel damage, with its abnormal elevation representing a novel mechanism in CSVD pathogenesis. Notably, BACE-1 has been proposed as a potential biomarker and therapeutic target for hypertensive CSVD (Zhou et al., 2022).

#### Vascular endothelial growth factor

Vascular endothelial growth factor (VEGF) was the first identified growth factor and is widely expressed in various cell types, including endothelial cells and SMCs (Fernezelian et al., 2025; Zheng et al., 2025). VEGF stimulates endothelial cell proliferation, inhibits apoptosis, supports neuronal cell survival, increases vascular permeability, and promotes angiogenesis (Zampetti et al., 2013; Balberova et al., 2021; Wu et al., 2024b). Its expression is regulated by hypoxia and is elevated in patients with acute ischemic stroke (Slevin et al., 2000; Lee et al., 2010; Dassan et al., 2012). In cerebral ischemic stroke studies, VEGF has been linked to CMBs, likely due to its role in compromising BBB integrity (Zhang et al., 2002; Ogata et al., 2019). Thus, serum VEGF levels are considered a potential biomarker for CMBs and are associated with the prognosis of cerebrovascular diseases (Lee et al., 2010; Yu et al., 2017), cardiovascular risk factors (Ferroni et al., 2012), and AD progression (Zhang et al., 2016). VEGF plays a key role in early vascular pathological alterations in white matter, with subsequent neuroinflammation potentially contributing to WMLs (Manukjan et al., 2023). Elevated VEGF levels have been correlated with the CSVD burden in older populations, highlighting its role in age-related CSVD progression (Dobrynina et al., 2020; Kapoor et al., 2021). Additionally, increased VEGF concentrations have been linked to hereditary forms of CSVD, including Fabry disease and CADASIL (Zampetti et al., 2013; Ping et al., 2019). Recent research has also explored the relationship between VEGF and cognitive function. Data from rat models suggest that activation of the STAT3/VEGF pathway may help reverse cognitive deficits associated with CSVD (Wang and Hu, 2018). Given its associations with CMBs, WMLs, CSVD progression, and cognitive impairment, VEGF has significant potential as a biomarker for CSVD.

#### Homocysteine

Homocysteine (HCY) plays a critical role in inflammation, atherosclerotic plaque formation, endothelial damage, arteriole injury, and oxidative stress. It contributes to endothelial dysfunction by directly damaging the endothelial lining or stimulating inflammatory responses, ultimately leading to white matter damage (Hassan et al., 2004a). HCY is recognized as a risk factor for ischemic white matter disease (Ma et al., 2010; Pavlovic et al., 2011). Vermeer et al. (2002) conducted a cross-sectional study showing that higher total HCY (tHCY) levels are associated with an increased risk of asymptomatic cerebral infarction and are significantly linked to the severity of periventricular and subcortical WMLs. Similarly, Naka et al. (2006) explored the relationship between tHCY levels and stroke-related leukosis or microbleeds, finding that elevated tHCY levels are associated with ischemic small artery disease but not with bleeding-prone small artery disease. HCY has also been linked to a higher risk of dementia (Miwa et al., 2016). Zhang et al. (2022b) identified elevated HCY as an independent risk factor for cognitive dysfunction in CSVD patients. A Mendelian randomization study further demonstrated that higher tHCY levels correlate with an increased CSVD burden, particularly LIs and brain volume loss, suggesting that reducing HCY levels may help lower the risk and slow CSVD progression (Cao et al., 2021). Although HCY may not be a definitive marker for CSVD, interventions to reduce its levels could potentially contribute to slowing CSVD progression.

### Neurovascular unit injury-related biomarkers

The neurovascular unit (NVU) is a complex structure composed of neurons, glial cells, and blood vessels (Ahn and Kim, 2024; Zhang et al., 2025). One of its primary functions is neurovascular coupling, which regulates blood flow and ensures the delivery of metabolic substrates, nutrients, and oxygen essential for proper brain function. Consequently, NVU damage can compromise the integrity of the BBB, potentially triggering or exacerbating the development of CSVD. This section explores CSVD-related biomarkers in terms of BBB breakdown, neuronal injury, and glial activation. Some biomarkers, such as neuron-specific enolase (NSE), glial fibrillary acidic protein (GFAP), and S100β, have shown potential as early screening markers. Notably, GFAP has been linked to CSVD severity. A summary of NVU biomarkers associated with CSVD is presented in **Additional Table 3**.

**Additional Table 3:** Association of neurovascular unit biomarkers with MRI characterization of CSVD

Blood biomarker	Neuroimaging marker	Study population	Reference
Angiopoietin-2	WMH	129 patients with end-stage renal disease aged > 65 yr	Bijkerk et al., 2022
Matrix metalloproteinase-9	WMH	80 individuals with WMH progression and 80 controls	Jimenez-Balado et al., 2021
Neurofilament light chain	WMH, atrophy	208 participants from the National University Hospital Memory Clinic and community in Singapore	Chong et al., 2023
Neuron-specific enolase	WMH	27 individuals with mild depressive episodes and 82 healthy controls over 60 yr of age	Polyakova et al., 2015
Glial fibrillary acidic protein	WMH	42 patients with sporadic CSVD	Huss et al., 2022
S100β	WMH	1563 participants from the Lothian Birth Cohort 1936 study	Cox et al., 2018
Triggering receptor expressed on myeloid cells 2	WMH	10 patients with Alzheimer's disease, 20 patients with SVD-CAA, and 46 patients with SVD-HTN	Tsai et al., 2021

CAA: Cerebral amyloid angiopathy; CSVD: cerebral small vessel disease; HTN: hypertension; WMH: white matter hyperintensity.

#### Blood–brain barrier breakdown

*Angiopoietin-2*

Angiopoietin-2 (Ang-2), a member of the angiogenin family, is predominantly expressed in VECs (David et al., 2010). In mouse models lacking pericytes, upregulation of specific proteins has been observed. Notably, proteins typically expressed during developmental phases, such as fibroblast growth factor binding protein 1 (Fgfbp1), and those associated with pathological angiogenesis, such as Ang-2, have been implicated in promoting cellular proliferation, dysregulation of the BBB at the arteriolar level, and diminished angiogenesis. Consequently, the expression levels of Fgfbp1 and Ang-2 may serve as critical biomarkers for BBB leakage in the progression of CSVD (Moretti et al., 2021). A study investigating biomarkers of cognitive impairment in elderly patients with end-stage renal disease revealed that Ang-2 levels are associated with imaging indicators of CSVD, particularly WMHs, and that Ang-2 levels exhibit an inverse correlation with cognitive function (Bijkerk et al., 2022).

*Matrix metalloproteinase-9*

Matrix metalloproteinases (MMPs) play two principal roles in inflammatory responses: they compromise the integrity of the BBB by hydrolyzing extracellular membrane (ECM) proteins and disrupt tight junctions, contributing to myelin degradation. When the BBB is compromised, MMPs from systemic circulation may infiltrate the brain, leading to elevated MMP levels in the cerebrospinal fluid (CSF). Among them, MMP-9 can degrade most ECM proteins and is involved in tissue inflammation and BBB disruption (Vafadari et al., 2016). MMP-9 levels have been associated with temporal changes or progression in WMHs (Bjerke et al., 2014; Jimenez-Balado et al., 2021), suggesting that MMP-9 plays a role in white matter damage. Additionally, animal studies indicate that MMP-9 is involved in the formation of LIs (Cayabyab et al., 2013). Elevated MMP levels contribute to inflammation and BBB disruption and may be linked to WMH and LI formation, thereby promoting the progression of CSVD.

#### Neuronal injury

*Neurofilament light chain*

Neurofilaments are intermediate filaments that primarily provide structural support in neuronal axons. Upon axonal injury, neurofilaments are released into the CSF and peripheral blood, making them potential biomarkers for neuronal damage. Numerous studies have emphasized the significance of neurofilament light chain (NFL) chain as a biomarker for axonal injury in various neurodegenerative diseases, including amyotrophic lateral sclerosis, multiple sclerosis, Parkinson’s disease, Huntington’s disease, brain injury, stroke, and dementia (Bridel et al., 2019; Chen et al., 2020; Xiang et al., 2022; Youssef et al., 2023). A cross-sectional study has identified an association between CSF NFL levels and the severity of WMHs, suggesting NFL’s potential involvement in CSVD (Kuhle et al., 2016). In studies on CADASIL, blood NFL levels have been correlated with neuroimaging markers of CSVD, potentially indicating disease progression (Chong et al., 2023; Wilms et al., 2024). Further research suggests that, compared to GFAP, NFL may have a stronger association with CSVD and could serve as a “bridge” linking CSVD to dementia (Wilms et al., 2024).

*Neuron-specific enolase*

NSE, an isoenzyme of glycolytic enolase, was initially believed to be restricted to nerve cells but was later found in neuroendocrine cells. It plays a role in neuronal energy metabolism, axoplasmic transport, neuroplasticity pathways, and cell survival and has been widely used as a marker of neuronal injury (Cheng et al., 2014). NSE has also been associated with various neurological diseases, including cerebral infarction (Cunningham et al., 1991), AD (Schmidt et al., 2014; Katayama et al., 2021), traumatic brain injury (Wang et al., 2018), and multiple sclerosis (Koch et al., 2015). Moreover, NSE levels are believed to correlate with disease severity. Koch et al. (2015) found that serum NSE levels were associated with disability and disease severity in patients with primary progressive multiple sclerosis. In ischemic stroke, serum NSE levels significantly correlate with infarct size and neurological severity (Wunderlich et al., 2004), making NSE a useful marker for predicting stroke severity and early functional outcomes (Zaheer et al., 2013). Polyakova et al. (2015) identified a positive correlation between serum NSE levels and the Fazekas score for WMLs in individuals with mild depression. Subsequent research has suggested that increased serum NSE levels may serve as an indicator of neuronal damage in individuals with mild neurocognitive impairment and could potentially be employed as an early biomarker for the development of WMLs (Polyakova et al., 2022).

#### Glial activation

*Glial fibrillary acidic protein*

Astrocytes play a dual role in either promoting or suppressing inflammation and neurodegeneration by regulating glutamate and ion homeostasis, cholesterol and sphingolipid metabolism, and the secretion of neurotoxic factors. They dynamically respond to environmental changes. Damage to arterioles within the deep white matter, coupled with endothelial dysfunction, leads to significant BBB disruption. This disruption allows tissue hypoxia and blood-borne cytokines to infiltrate the perivascular brain parenchyma, activating microglia and promoting astrocyte proliferation, ultimately resulting in axonal injury. GFAP is predominantly expressed by astrocytes activated during neuroinflammatory responses and serves as a key indicator of glial cell activation. Specifically, GFAP is recognized as a biomarker for neuroinflammatory responses in WMLs (Chong et al., 2024). A prospective study has found that serum GFAP levels correlate with neurocognitive function and clinical severity in patients with CSVD, underscoring its potential as a biomarker for this condition (Huss et al., 2022). Furthermore, research by Gattringer et al. (2023) has demonstrated that GFAP functions as a selective biomarker for CSVD, exhibiting sensitivity to acute tissue alterations but limited efficacy in detecting chronic changes. Targeting astrocytic signaling pathways may offer promising therapeutic strategies for mitigating the risk of vascular dementia (Hase et al., 2018). Consequently, GFAP, as an indicator of astrocyte activation and reactive gliosis, holds potential as a novel biomarker for assessing CSVD severity (Hase et al., 2018; Chen et al., 2020; Wilms et al., 2024).

*S100*β *protein*

S100β, a member of the S100 protein family, regulates various cellular responses through calcium signaling pathways. It is secreted by oligodendrocytes and astrocytes and plays a role in cell growth, energy metabolism, and inflammatory responses (Zhang et al., 2024a). S100β exhibits concentration-dependent physiological effects: at nanomolar concentrations, it promotes neuroprotection and neuronal growth, whereas at micromolar concentrations, it stimulates the production of pro-inflammatory cytokines (Cox et al., 2018). As a biomarker of cell injury in the central nervous system, sustained elevation of S100β may indicate ongoing glial damage or chronic inflammatory activation (Zhang et al., 2024a). Notably, S100β can cross the BBB and be detected in peripheral circulation, making it a useful marker for brain injury (Kleindienst et al., 2010). It has been linked to various neurological conditions, including traumatic brain injury (Ingebrigtsen and Romner, 2002; Wiesmann et al., 2010), stroke (James et al., 2009; Brouns et al., 2010), and neurodegenerative disorders (Peskind et al., 2001; James et al., 2009; Chaves et al., 2010). Research by Cox et al. (2018) has examined the relationship between serum S100β levels and MRI findings in older individuals, identifying S100β as a potential biomarker for white matter aging. Additionally, S100β levels show a positive correlation with age (Schroeter et al., 2011). Given that CSVD is an age-dependent condition, current research suggests a link between CSVD-related cognitive impairment and serum S100β levels. Consequently, early detection of serum S100β may be critical for diagnosing cognitive impairment associated with CSVD (Wang et al., 2017).

*Triggering receptor expressed on myeloid cells 2*

Triggering receptor expressed on myeloid cells 2 (TREM2), an innate immune receptor belonging to the immunoglobulin superfamily, is predominantly expressed on microglial cells in the brain. TREM2 plays a vital role in promoting the clearance of damaged myelin by microglia and supports myelin regeneration by oligodendrocytes (Poliani et al., 2015). TREM2 is implicated in the pathogenesis of AD, where its dysfunction leads to reduced efficiency in Aβ clearance (Xue and Du, 2021). Given this involvement, TREM2 has been identified as a biomarker for neurodegenerative conditions (Suarez-Calvet et al., 2016). Additionally, plasma TREM2 levels correlate with CRP, highlighting its role as an indicator of peripheral inflammation (Bekris et al., 2018). A cross-sectional study assessing plasma TREM2 concentrations in patients with AD and CSVD, alongside imaging markers and brain amyloid burden, found a positive association between soluble TREM2 and WMHs. These findings suggest that soluble TREM2 may serve as a promising biomarker for white matter damage associated with CSVD (Tsai et al., 2021).

### Coagulation-related biomarkers

The regulation of coagulation and fibrinolysis is a crucial physiological function of VECs. Damage to VECs exposes ECM-associated proteins, such as collagen, which promotes the binding of circulating von Willebrand factor (vWF) to collagen. This interaction anchors vWF to the damaged vessel, initiating its activation. This activation leads to platelet adhesion, aggregation, and the recruitment of coagulation factors, ultimately triggering the coagulation cascade. An increasing body of research has highlighted the significant role of coagulation and fibrinolysis in the pathogenesis and progression of CSVD.

Interestingly, a prospective study exploring the predictive value of endothelial activation markers for the progression of WMHs found no support for the role of coagulation activation in the early progression of CSVD. However, the study underscores the potential importance of coagulation mechanisms in explaining the occurrence of acute LIs in patients already diagnosed with CSVD (Markus et al., 2005).

A summary of the NVU biomarkers associated with CSVD features is provided in **Additional Table 4**.

**Additional Table 4:** Association of coagulation biomarkers with magnetic resonance imaging features of CSVD

Blood biomarker	Neuroimaging marker	Study population	Reference
Fibrinogen	WMH and lacunar infarction	123 participants, including 49 with lacunar stroke, 48 with vascular dementia, and 26 with vascular Parkinson's syndrome.	Staszewski et al., 2018b
von Willebrand factor	WMH and lacunar infarction	296 patients with CSVD aged ≥ 60 yr	Wiseman et al., 2014; Sun et al., 2021
CD40 ligand and platelet-selectin (CD62P)	WMH and lacunar infarction	960 participants aged > 35 yr	Zhang et al., 2022a
Biomarker clustering	WMH	494 participants aged > 50 yr from the Heart Brain Connection study	Kuipers et al., 2022

CSVD: Cerebral small vessel disease; WMH: white matter hyperintensity.

#### Fibrinogen

Fibrinogen (FIB) is a key biomarker of systemic hypercoagulability and inflammation, and is considered a reliable indicator of BBB damage (Staszewski et al., 2018a). Elevated FIB levels are associated with increased thrombus formation, reduced cerebral blood flow, and a heightened risk of cardiovascular events (Ernst and Resch, 1993; Fibrinogen Studies Collaboration et al., 2005). High FIB levels have also been linked to white matter damage, LIs, and the progression of CSVD (Schmidt et al., 1997; Wada et al., 2011; Staszewski et al., 2018a). Moreover, increased FIB levels correlate with cognitive impairment (Marioni et al., 2009; Rafnsson et al., 2010). Therefore, FIB holds promise as a biomarker for CSVD and may also serve as a predictor of CSVD-related cognitive decline.

#### von Willebrand factor

Circulating vWF in the blood interacts with collagen, anchoring itself to damaged blood vessels. This interaction triggers platelet activation by binding to the GPIb receptor, leading to platelet adhesion, aggregation, and the recruitment of coagulation factors, thereby initiating the coagulation cascade. A meta-analysis of lacunar stroke, non-lacunar ischemic stroke, and non-stroke cases examined coagulation-related biomarkers, fibrinolysis, endothelial dysfunction, and inflammation (Wiseman et al., 2014). The analysis found that specific biomarkers, including vWF, were significantly elevated in individuals with LIs compared to non-stroke individuals. However, vWF expression was notably lower in lacunar stroke cases than in other forms of ischemic stroke. Additionally, a cross-sectional study identified an association between elevated vWF levels and the presence of WMHs (Sun et al., 2021). Another study found a similar relationship between high vWF levels and periventricular WMH, rather than deep WMH (Vilar-Bergua et al., 2016). Consequently, the potential of vWF as a biomarker for CSVD warrants further investigation.

#### CD40 ligand and platelet selectin

Platelet selectin (CD62p) is a marker of platelet activation. Upon stimulation by lipopolysaccharides via the TLR4 receptor, platelets express CD62p on their surface and secrete CD40 ligand (CD40L), along with other pro-inflammatory mediators. This platelet activation results in an upregulation of CD40L and CD62p expression. Both CD40L and CD62p have been implicated in the pathogenesis of WMHs and LIs (Zhang et al., 2022a). Additionally, Oberheiden et al. (2010) found that the expression of CD40L and CD62p on platelets is elevated in patients with CSVD, which may activate the coagulation cascade and facilitate the development of CSVD.

#### Clustering of coagulation-related biomarkers

While numerous studies have explored the relationship between individual blood biomarkers and CSVD, a 2022 study took a novel approach by examining the clustering of coagulation-related biomarkers and their association with CSVD (Kuipers et al., 2022). This research provides the first comprehensive assessment of the relationship between a broad range of blood-based biomarkers and CSVD manifestations. Although limited by its cross-sectional design, which precludes establishing a causal link between these biomarkers and CSVD progression, the findings still suggest a significant association between coagulation abnormalities and the development of CSVD.

### Metabolism-related biomarkers

#### 25-hydroxyvitamin D

High levels of vitamin D can upregulate multiple neurotrophic factors, such as NT-3, GDNF, and BDNF, supporting the growth, maintenance, and survival of neuronal cells. Vitamin D is also crucial for maintaining the structural integrity of brain tissues (Sun and Tian, 2021). 25-Hydroxyvitamin D (25(OH)D) is widely recognized as the optimal indicator of vitamin D status and serves as a biomarker for vitamin D levels in the body. Additionally, 25(OH)D is associated with vascular risk factors (Watson et al., 1997; Forman et al., 2007; Kassi et al., 2013), CVDs (Al Mheid et al., 2011; Brondum-Jacobsen et al., 2013), and cognitive impairment (Littlejohns et al., 2014; Chai et al., 2019). The vascular protective role of 25(OH)D may be attributed to its ability to activate endothelial cells and alter the structure of microvessels (Borges et al., 1999; Pilz et al., 2011). Deficiency in 25(OH)D can disrupt macrophage and lymphocyte activity in atherosclerotic plaques, leading to enhanced inflammatory responses in the arterial walls (Balden et al., 2012). In 2015, Chung et al. identified an association between 25(OH)D levels and several neuroimaging markers of CSVD, including LIs, WMH, and deep CMBs, suggesting a potential correlation between 25(OH)D levels and chronic brain damage linked to CSVD. A subsequent study by Feng et al. (2019) further demonstrated a correlation between 25(OH)D levels and the overall MRI burden of CSVD. While previous studies have suggested a connection between low 25(OH)D levels and a high CSVD burden, the causal relationship remains unclear. A Mendelian randomization study by Lee et al. (2023) analyzed the causal relationship between 25(OH)D levels and CSVD-related phenotypes. Their findings indicate no significant causal effects of 25(OH)D levels on LIs, WMH, or PVSs, but they report a reverse causal relationship with CMBs. This suggests that low 25(OH)D levels are not a direct cause of CSVD; rather, a high CSVD burden may lead to a reduction in 25(OH)D levels. Consequently, further research is needed to determine whether 25(OH)D could serve as a promising biomarker for CSVD.

#### Cystatin C

Cystatin C (Cys-C), the principal endogenous inhibitor of cysteine proteases, plays a crucial role in maintaining the normal physiological architecture of blood vessels. It is also considered a key biomarker for assessing kidney function impairment (Van Den Noortgate et al., 2002). Notably, changes in kidney function may indicate an increased risk of cerebrovascular diseases (Go et al., 2004; Riverol et al., 2015). An imbalance between Cys-C and cysteine protease expression can lead to vascular wall remodeling and neuroinflammation (Singh et al., 2007), both of which are significant pathophysiological mechanisms in CSVD. A cross-sectional study investigating the association between Cys-C, cognitive impairment, and CSVD in older community residents revealed a close relationship between Cys-C levels and subclinical CSVD in this population. Additionally, Cys-C serves as an independent risk factor for LIs and WMH (Wada et al., 2010). Studies have consistently shown a positive correlation between higher Cys-C levels and cognitive impairment (Yaffe et al., 2008; Riverol et al., 2015). Guoxiang et al. (2018) proposed that Cys-C could serve as a biomarker for white matter damage. Furthermore, Cys-C has been identified as the most sensitive indicator of CMB severity (Oh et al., 2014). In contrast, Yao et al. (2022) posited that Cys-C is linked to LIs and WMH but not to CMBs. Therefore, further investigation is needed to clarify whether a definitive association exists between Cys-C levels and CMBs. Although a Mendelian randomization study has established no causal relationship between Cys-C levels and CVDs, its potential role in predicting disease progression remains significant (van der Laan et al., 2016). Thus, Cys-C continues to hold promise as a biomarker for CSVD (van der Laan et al., 2016; Yang et al., 2017; Yao et al., 2022).

#### Phosphates

Abnormally elevated phosphate levels in the blood are commonly observed in individuals with underlying conditions such as chronic kidney disease. Chung et al. (2019) identified high phosphate levels as a contributor to vascular calcification and stiffening, which may lead to the development of CSVD and correlate with the severity of WMH. They suggest that elevated circulating phosphate could serve as a potential therapeutic target for CSVD. Dietary modifications or the use of phosphate binders designed to reduce oral phosphate intake may help lower phosphate levels in the body, thereby contributing to the treatment or alleviation of CSVD.

Due to the complexity of metabolic processes in humans, establishing a causal relationship between metabolism-related biomarkers and the development of CSVD remains challenging. However, these biomarkers can still serve as indicators of disease severity. A summary of metabolism-related biomarkers associated with CSVD features is presented in **Additional Table 5**.

**Additional Table 5:** Association of metabolism-related biomarkers with magnetic resonance imaging features of cerebral small vessel disease

Blood biomarker	Neuroimaging marker	Study population	Reference
25-Hydroxyvitamin D	Cerebral microbleeds	Mendelian Randomization study	Lee et al., 2023
Cystatin C	WMH, lacunar infarcts, and perivascular space	3061 participants average aged 61.2 ± 6.7 yr	Yao et al., 2022
Phosphates	WMH	186 participants aged > 50 yr from the I-Lan Longitudinal Aging Study	Chung et al., 2019

WMH: White matter hyperintensity.

### Aging-related blood biomarkers

#### Vascular endothelial growth factor receptor-2

VEGF is associated with aging, and its receptor, VEGFR2, plays a crucial role in regulating cell fate. VEGFR2 is involved in angiogenesis and increased vascular permeability, with its expression levels correlating with age (Ahmed-Jushuf et al., 2016). While there is a lack of direct correlation studies establishing a relationship between VEGF levels and CSVD, the association between CSVD, VEGF levels, and cognitive function suggests that further investigation into the relationship between VEGFR2 levels and CSVD could provide valuable insights into the underlying mechanisms of the condition.

#### Nicotinamide adenine dinucleotide phosphate oxidase

Nicotinamide adenine dinucleotide phosphate (NADPH) oxidase contributes to vascular damage by producing excessive superoxide, which scavenges NO or inhibits endothelial nitric oxide synthase. Experimental studies on aged rats with impaired eNOS-dependent vasodilation of cerebral arterioles have demonstrated that treatment with NADPH oxidase inhibitors can restore vasodilatory function (Mayhan et al., 2008). Aging has been shown to promote the activation of NADPH oxidase in cerebral arterioles, and evidence suggests that NADPH oxidase activation plays a key role in the pathogenesis of CSVD (McCarty, 2015). Intracellularly, free bilirubin acts as an inhibitor of NADPH oxidase (Lanone et al., 2005), and serum bilirubin levels are inversely correlated with the risk of LIs and WMLs (Li et al., 2014). Oxidative stress plays a crucial role in the pathogenesis of CSVD (Wu et al., 2024a), and NADPH, as a major source of reactive oxygen species (ROS), is involved in this process. Therefore, NADPH oxidase may serve as a potential biomarker for CSVD, and inhibition of its activity could offer therapeutic benefits in improving CSVD outcomes (McCarty, 2015). However, it is important to note that oxidative stress is not a mechanism unique to CSVD, which limits the specificity of NADPH oxidase as a biomarker.

#### Klotho

Klotho protein, encoded by the Klotho gene, is an anti-aging protein primarily expressed in distal tubular cells of the kidneys, the choroid plexus, and brain parenchyma. In VECs, Klotho plays a critical role in regulating NO, maintaining calcium homeostasis, and inhibiting oxidative stress and inflammatory responses (Chang et al., 2005; Rakugi et al., 2007; Maekawa et al., 2009; Drueke and Massy, 2013). A deficiency in the Klotho gene is associated with reduced lifespan, while its overexpression is linked to extended longevity (Kurosu et al., 2005; Semba et al., 2011). Furthermore, decreased plasma Klotho levels are correlated with the severity of WMHs and cognitive decline (Kuriyama et al., 2018). Studies in patients with acute stroke have revealed a negative correlation between plasma Klotho levels and the presence, burden, and progression of CSVD (Woo et al., 2019). However, Klotho levels are influenced by multiple factors, including oxidative stress and inflammatory responses, which may limit its utility as a biomarker.

## Cerebrospinal Fluid–Based Biomarkers

**Figure 3:**
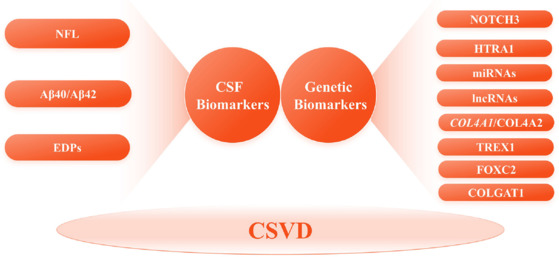
CSVD-related CSF biomarkers and genetic biomarkers. Aβ: Amyloid-β; COL4A1: collagen type IV alpha 1 chain; COL4A2: collagen type IV alpha 2 chain; COLGAT1: collagen beta (1-O) galactosyltransferase 1; CSVD: cerebral small vessel disease; EDPs: elastin-derived peptides; FOXC1: forkhead box C1; FOXC2: forkhead box C2; lncRNAs: long non-coding RNAs; miRNAs: microRNAs; NFL: neurofilament light chain; NOTCH3: neurogenic locus notch homolog protein 3; TREX1: three prime repair exonuclease 1.

After NVU injury, biomolecules released from the damaged tissue not only enter the peripheral circulation but also are present in the CSF, including MMP-9 (Bjerke et al., 2014), FIB (McAleese et al., 2019), NFL, Aβ_40_/Aβ_42_, and EDP (**Figure 3**). Studies have shown that some of these molecules are significantly more concentrated in the CSF than in serum, which may make them more sensitive to detection. However, obtaining relevant CSF markers is more challenging due to the invasive nature of lumbar puncture, compared to the less invasive methods used for blood and imaging markers. A summary of the CSF biomarkers associated with CSVD features is shown in **Additional Table 6**.

**Additional Table 6:** Association of cerebrospinal fluid biomarkers with magnetic resonance imaging features of cerebral small vessel disease

Biomarker	Neuroimaging marker	Study population	Reference
Matrix metalloproteinase-9	WMH	46 non-demented older patients with cerebral small vessel disease	Bjerke et al., 2014
Fibrinogen	WMH	42 human postmortem brain donors	McAleese et al., 2019
Neurofilament light chain	WMH	22 patients with Alzheimer's disease, 9 patients with small vessel disease, and 20 healthy controls	Sjogren et al., 2001
Amyloid-340/42	WMH	112 participants from Alzheimer's Disease Neuroimaging Initiative	Scott et al., 2016
Elastin peptide	lacunar infarcts	80 patients with acute ischemic stroke and 15 controls	Tzvetanov et al., 2008

WMH: White matter hyperintensity.

### Neurofilament light chain

NFL in both serum and CSF has been recognized as a biomarker for neurological disorders (Wilms et al., 2024). Notably, the concentration of NFL in CSF is significantly higher than in serum, with serum NFL exhibiting lower sensitivity compared to CSF NFL (Kartau et al., 2022). As a non-specific biomarker of aging and white matter integrity, NFL levels in CSF increase with advancing age, providing an indication of the burden associated with CSVD (Sjogren et al., 2001; Meeker et al., 2022).

### Amyloid-β40/42

The deposition of Aβ proteins is widely recognized as a key pathological feature of AD, with research establishing a strong association between Aβ and CAA (Zhu et al., 2020). CAA, a common form of SVD, is primarily characterized by the accumulation of Aβ_40_ in small blood vessels (Theodorou et al., 2021). Recently, a study using a transgenic rat model of CAA (rTg-DI) showed that Aβ_40_ levels are reduced in both CSF and blood, with this reduction occurring prior to the manifestation of CMBs. Based on these findings, researchers propose that CSF and blood Aβ_40_ levels may serve as early biomarkers for the onset of CAA (Zhu et al., 2020). Furthermore, in a cohort of cognitively normal older individuals with low-to-moderate vascular risk factors, baseline amyloid burden has been associated with greater accumulation of WMHs over the following 2 years (Scott et al., 2016). While Aβ proteins are recognized as significant biomarkers for AD, they also play a crucial role in CAA, making them vital for the early detection of this condition.

### Elastin-derived peptides

Elastin, a major component of the vascular ECM, produces elastin-derived peptides (EDPs) through its degradation. Under normal conditions, EDPs are present in trace amounts in both blood and CSF (Tzvetanov et al., 2008). Elastin degradation is recognized as a key factor in the development of vascular wall abnormalities associated with aging, AS, and diabetic vasculopathy (Baydanoff et al., 1987). As a result, EDPs are considered markers of vascular injury. A comparative study of CSF EDP levels among patients with lacunar stroke, large vessel stroke, and recurrent LIs found that patients with LIs due to CSVD exhibited significantly higher CSF EDP levels compared to those with large artery strokes. This suggests that small vessel AS may exacerbate elastin degradation to a greater extent than large vessel AS (Tzvetanov et al., 2008).

## Cerebral Small Vessel Disease–Related Genetic Biomarkers

### Neurogenic locus notch homolog protein 3

CADASIL is a hereditary form of CSVD, primarily caused by mutations in the neurogenic locus notch homolog protein 3 (*NOTCH3*) gene. This gene encodes a critical single-pass transmembrane receptor that is essential for the maturation and differentiation of vascular SMCs and the maintenance of vascular integrity. Mutations in the *NOTCH3* gene lead to the accumulation of granular osmiophilic material and aggregation of the NOTCH3 extracellular domain (*NOTCH3*(ECD)) (Rutten et al., 2016). The increased aggregation of *NOTCH3*(ECD) damages the ECM of vascular walls, compromising the integrity of small blood vessels and contributing to the development of CSVD (Tan et al., 2017).

### HTRA1

The genetic basis of cerebral autosomal recessive arteriopathy with subcortical infarcts and leukoencephalopathy (CARASIL) is not yet fully understood. This condition is characterized by ischemic, non-hypertensive CSVD, often accompanied by alopecia and cervical spondylosis. It is hypothesized that mutations in the *HTRA1* gene underlie CARASIL. The *HTRA1* gene encodes a serine protease that plays a crucial role in maintaining vascular integrity (Ito et al., 2018). Emerging evidence also suggests that mutations in the *HTRA1* gene contribute to the development of CADASIL, with heterozygous *HTRA1* mutations potentially implicated in the pathogenesis of CSVD (Liu et al., 2020; He et al., 2023).

### MicroRNAs

MicroRNAs (miRNAs) are a class of small non-coding RNA molecules, typically 19 to 24 nucleotides in length, that regulate gene expression by inhibiting translation and promoting the degradation of target mRNAs through binding to complementary regions of the transcripts (Bartel, 2009). Due to their relative stability in the circulatory system, miRNAs have been proposed as biomarkers for various diseases. Specifically, miRNAs have been explored as biomarkers for detecting cancers (Mitchell et al., 2008; Schwarzenbach, 2017), CVDs (Navickas et al., 2016; Almaghrbi et al., 2023), and neurodegenerative disorders (Sheinerman and Umansky, 2013; Sheinerman et al., 2017). Prabhakar et al. (2017) proposed that plasma miRNAs could differentiate individuals with CSVD from healthy controls, suggesting that miRNAs may serve as diagnostic biomarkers for CSVD. Notably, miR-29a has been shown to be positively correlated with WMH scores (Bijkerk et al., 2022).

### Long non-coding RNAs

Long non-coding RNAs (lncRNAs), a class of non-coding RNAs longer than 200 nucleotides, play critical roles in various biological functions such as chromatin remodeling, transcriptional regulation, and post-translational modification of proteins (Boon et al., 2016; Gao et al., 2024; Zeng et al., 2024). Emerging evidence has highlighted the differential expression of lncRNAs as key indicators for CVDs and neurodegenerative disorders (Riva et al., 2016; Chen et al., 2021). For example, the lncRNA NF-κB interacting long noncoding RNA (*NKILA*) has been identified for its interaction with the pro-inflammatory transcription factor NF-κB, inhibiting its transcriptional activity. This mechanism not only mitigates inflammatory responses and oxidative stress but also provides neuroprotection in cerebral ischemia (Gao et al., 2021). A prospective study by Lapikova-Bryhinska et al. (2024) explored the relationship between lncRNAs and ischemic stroke risk, showing that *NKILA* confers protective effects against cerebral ischemia. This underscores the potential of *NKILA* in the diagnosis and treatment of vascular dementia. Additionally, *NKILA* is associated with LIs and could serve as a promising biomarker for the prevention and treatment of aging-related diseases. Furthermore, metastasis-associated lung adenocarcinoma transcript 1 (*MALAT1*), a highly conserved lncRNA located on chromosome 11q13, was one of the first lncRNAs identified to play a functional role in cancers (Malakar et al., 2019). Elevated levels of *MALAT1*, induced by high glucose, have been shown to enhance apoptotic potential in microvascular endothelial cells by regulating TPR expression via miR-7641, thereby exacerbating neurological dysfunction associated with CSVD (Che et al., 2021). Additionally, plasma exosomal lncRNAs have promising diagnostic potential for WMHs (Xu et al., 2023), and circulating *MALAT1* has been identified as a potential biomarker for SVD (Yan et al., 2018).

### Collagen type IV alpha 1 chain/collagen type IV alpha 2 chain

The collagen type IV alpha 1 chain (*COL4A1*) and collagen type IV alpha 2 chain (*COL4A2*) genes, located in tandem on chromosome 13q34, share a common bidirectional promoter and encode collagen type IV (*COL4*). COL4 is a major component of the vascular basement membrane, playing a crucial role in maintaining the stability and function of the basement membrane (Poschl et al., 2004). Mutations in the *COL4A1* gene, which encodes *COL4*, can disrupt the normal formation of functional collagen, resulting in small vessel abnormalities, increased vulnerability of arterioles, and diffuse microangiopathy (Gould et al., 2006; Volonghi et al., 2010). Moreover, studies have shown that missense mutations in the *COL4A1*/*COL4A2* genes are not only implicated in rare familial forms of CSVD (Kuuluvainen et al., 2021) but also associated with sporadic cases of CSVD (Rannikmae et al., 2015; Liang et al., 2019).

### Three prime repair exonuclease 1

Three prime repair exonuclease 1 (*TREX1*) is the most abundant 3′–5′ DNA exonuclease in mammals, and mutations in this gene are known to cause retinal vasculopathy with cerebral leukodystrophy (RVCL), a hereditary form of SVD (Chauvin et al., 2024). RVCL has been proposed as a monogenic model for vascular dementia (de Boer et al., 2022). *TREX1* is highly expressed in oligodendrocytes, which may explain why frameshift mutations in this gene lead to white matter degeneration (Saito et al., 2019). Additionally, *TREX1* plays a critical role in cerebral ischemic injury (Kothari et al., 2018). Pelzer et al. (2013) suggest that *TREX1* mutations may contribute to early-onset cerebrovascular diseases, and the mechanisms underlying these mutations may offer novel insights into the pathogenesis of cerebrovascular diseases.

### Forkhead box C1/forkhead box C2

The forkhead box C1 (FOXC1) and forkhead box C2 (FOXC2) genes are located on chromosomes 6 (6p25) and 16 (16q22-q24), respectively. They are part of the forkhead (fox) family of transcription factors, which are closely associated with embryonic development and are essential for the development of the cardiovascular system (Kume, 2009). Both genes share similar regulatory functions. *FOXC1*, in particular, plays a critical role in maintaining the integrity of the vascular basement membrane (Skarie and Link, 2009). Mutations in *FOXC1* have been shown to disrupt platelet-derived growth factor signaling and compromise vascular stability. Furthermore, MRI scans of patients with *FOXC1* mutations often reveal imaging features characteristic of CSVD (French et al., 2014). The *PITX2*-encoded transcription factor interacts with *FOXC1*, and evidence suggests that *FOXC1* and *PITX2* may jointly contribute to CSVD through a shared molecular pathway (French et al., 2014; Neurology Working Group of the Cohorts for et al., 2016).

### Collagen beta (1-O) galactosyltransferase 1

Collagen beta (1-O) galactosyltransferase 1 (*COLGALT1*) is an enzyme widely expressed in human tissues, with strong activity toward COL4. Reduced activity of *COLGALT1* impairs the secretion of COL4. Evidence suggests that variations in *COLGALT1* contribute to abnormalities in cerebral small vessels through a shared molecular pathogenic mechanism involving *COL4A1*/*COL4A2*-related diseases (Miyatake et al., 2018).

Genetic biomarkers are highly specific and significant for early diagnosis, prognostic assessment, and therapeutic target exploration (**Figure 3**). However, it is important to note that some CSVD-related genetic biomarkers, including *NOTCH3*, *COL4A1*/*COL4A2*, *TREX1*, and *COLGALT1*, are not exclusively linked to CSVD development. A summary of gene-related biomarkers associated with CSVD features is shown in **Additional Table 7**.

**Additional Table 7:** Association of gene-related biomarkers with CSVD genetic disorders

Biomarker	Neuroimaging marker	Disease	Reference
NOTCH3	WMH, small subcortical infarct	CADASIL	Rutten et al., 2016
HTRA1	WMH, small subcortical infarct	CARASIL/CADASIL	Liu et al., 2020
miRNAs	WMH	CSVD	Bijkerk et al., 2022
lncRNA	WMH, lacunar infarcts	CSVD	Xu et al., 2023; Lapikova-Bryhinska et al., 2024
COL4A1/COL4A2	CMBs	CSVD	Rannikmae et al., 2015
TREX1	WMH	Retinal vasculopathy with cerebral leukoencephalopathy	Saito et al., 2019
FOXC1/FOXC2	WMH, lacunar infarcts, PVS	CSVD	French et al., 2014

CADASIL: Cerebral autosomal dominant arteriopathy with subcortical infarcts and leukoencephalopathy; CARASIL: cerebral autosomal recessive arteriopathy with subcortical infarcts and leukoencephalopathy; COL4A1: collagen type IV alpha 1 chain; COL4A2: collagen type IV alpha 2 chain; CSVD: cerebral small vessel disease; FOXC1: forkhead box C1; FOXC2: forkhead box C2; lncRNAs: Long non-coding RNAs; miRNAs: microRNAs; NOTCH3: neurogenic locus notch homolog protein 3; TREX1: three prime repair exonuclease 1; WMH: white matter hyperintensity.

## Limitations

This review has several limitations. There is no in-depth discussion of the potential mechanisms underlying these biomarkers. Additionally, most of the studies reviewed are cross-sectional and lack long-term follow-up data, which makes it challenging to assess the relationship between dynamic changes in biomarkers and disease prognosis.

## Conclusion

This review provides an overview of CSVD-related biomarkers from blood, CSF, and genetic perspectives. It integrates contemporary insights into the potential mechanisms underlying CSVD and explores the relationships between these biomarkers and imaging features, as well as their associations with cognitive impairment. The aim is to establish a framework for the future identification of specific biomarkers for CSVD. Research on these biomarkers holds promising potential for advancing early diagnostic and therapeutic strategies, while also deepening our understanding of the underlying pathogenesis. However, most existing studies are cross-sectional, and none have identified biomarkers with sufficient specificity. Longitudinal investigations are essential to validate the reliability and clinical utility of these biomarkers.

Plasma-based biomarkers are accessible, minimally invasive, and cost-effective, making them suitable for the early identification and monitoring of CSVD. However, specific biomarkers, such as NFL, demonstrate enhanced sensitivity in CSF analysis. It is crucial to recognize that these biomarkers may exhibit limited specificity. In contrast, genetic biomarkers offer high specificity and play a key role in identifying therapeutic targets.

This review emphasizes the challenges posed by the diversity and complexity of CSVD pathogenesis, which complicate the reliance on a single biomarker for indicating its onset and progression. As a result, the potential value of using multiple biomarkers in combination, such as biomarker clusters, warrants further experimental investigation in future research.

## Additional files:

***Additional Table 1:*** *Associations of systemic inflammation biomarkers with MRI features of CSVD.*

***Additional Table 2:*** *Association of endothelial injury biomarkers with MRI features of CSVD.*

***Additional Table 3:*** *Association of neurovascular unit biomarkers with MRI characterization of CSVD.*

***Additional Table 4:*** *Association of coagulation biomarkers with magnetic resonance imaging features of CSVD.*

***Additional Table 5:*** *Association of metabolism-related biomarkers with magnetic resonance imaging features of cerebral small vessel disease.*

***Additional Table 6:*** *Association of cerebrospinal fluid biomarkers with magnetic resonance imaging features of cerebral small vessel disease.*

***Additional Table 7:*** *Association of gene-related biomarkers with CSVD genetic disorders.*

## Data Availability

*All relevant data are within the paper and its Additional files*.
